# Population transcriptomics with single-cell resolution: A new field made possible by microfluidics: A technology for high throughput transcript counting and data-driven definition of cell types

**DOI:** 10.1002/bies.201200093

**Published:** 2012-12-27

**Authors:** Charles Plessy, Linda Desbois, Teruo Fujii, Piero Carninci

**Affiliations:** 1 RIKEN Omics Science Center, Suehiro-chôTsurumi-ku, Yokohama, Japan; 2 LIMMS/CNRS-IIS UMI 2820, University of TokyoMeguro-ku, Tokyo, Japan; 3 Institute of Industrial Science, University of TokyoMeguro-ku, Tokyo, Japan

**Keywords:** high throughput, microfluidics, population, single cells, transcript counting

## Abstract

Tissues contain complex populations of cells. Like countries, which are comprised of mixed populations of people, tissues are not homogeneous. Gene expression studies that analyze entire populations of cells from tissues as a mixture are blind to this diversity. Thus, critical information is lost when studying samples rich in specialized but diverse cells such as tumors, iPS colonies, or brain tissue. High throughput methods are needed to address, model and understand the constitutive and stochastic differences between individual cells. Here, we describe microfluidics technologies that utilize a combination of molecular biology and miniaturized *labs on chips* to study gene expression at the single cell level. We discuss how the characterization of the transcriptome of each cell in a sample will open a new field in gene expression analysis, *population transcriptomics*, that will change the academic and biomedical analysis of complex samples by defining them as quantified populations of single cells.

## Introduction

**Figure 1:**
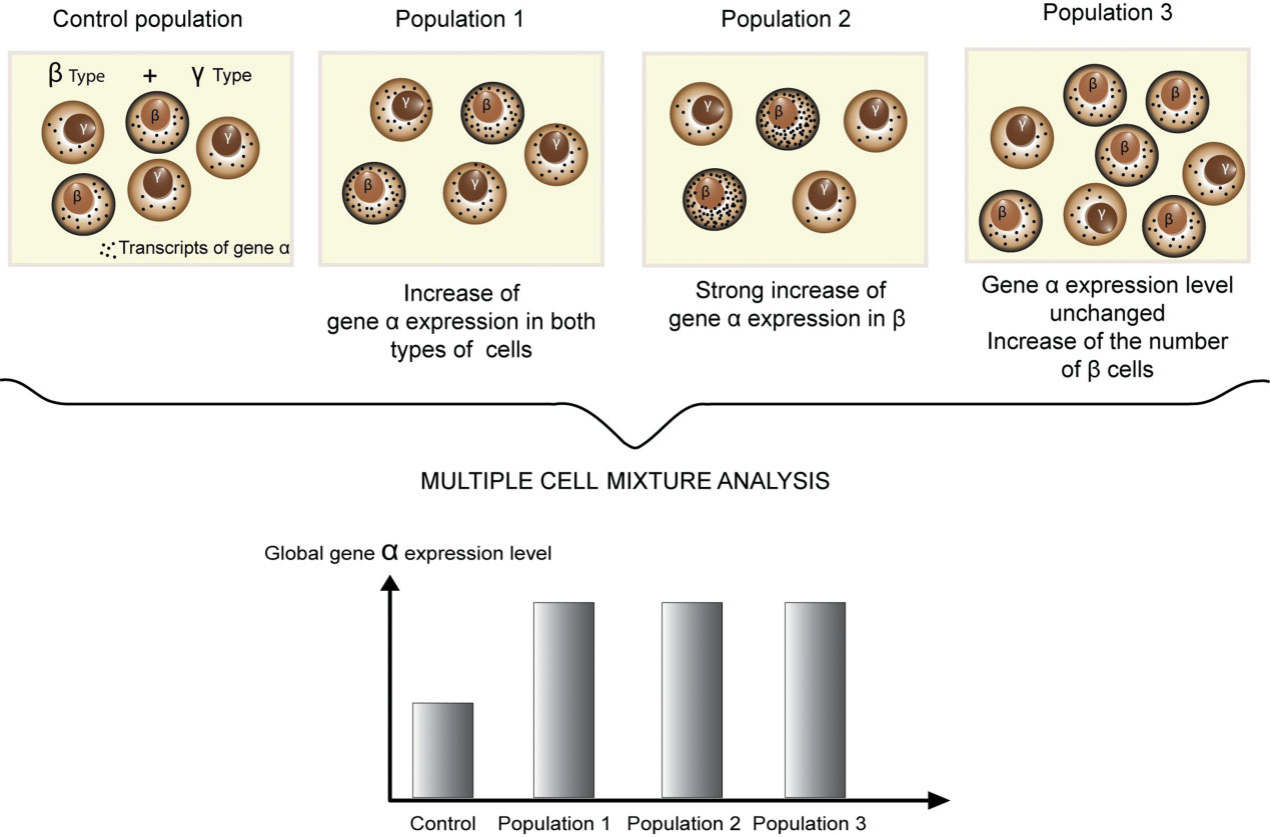
The limits of cell mixture analysis. If an increase in gene expression is detected in a mixture of cells, is it because of: (1) a change in all cells of the population? (2) a stronger change in one cell type, the other type being non-responsive? or (3) proliferation of a highly-expressing cell type?

Even for cells that are, in appearance, functionally homogeneous, for instance neurons sharing shape, neurotransmitter, and spatial location, it is not proven that these resemblances correlate at the transcriptome level. Concomitantly, very specific molecular markers are also scarce (see Okaty et al. 1 for review). Cell mixtures are of limited power for analyzing the dynamics of gene expression, as one cannot distinguish between a change of the same amplitude in all the cells, a change taking place in a subset of the cells, or a change in the composition of the cell population (Fig. 1). Parallel analyses of single cells can resolve these questions, and change our view of cell populations. For example, showing phenotypic variations between individuals even when they are strongly related, such as cells growing together in cell culture 2, 3. These single-cell analyses are based mainly on transcript imaging 4, PCR 5, or transcriptome analysis by microarray, and more recently, sequencing 6.

Some of these pioneering works have established that initiation of transcription can occur as *bursts*, for instance in Chinese hamster ovary cells 7, *Dictyostelium* 8, *Saccharomyces cerevisiae* 9, etc. During these bursts, multiple copies of a transcript are generated in a short interval followed by a period of rest that can be long when compared with the RNA's half-life. Thus, in a population of cells where a given protein is expressed, some cells may actually contain no corresponding transcript. Combined with other sources of variation like the cell cycle, these biological mechanisms create a large collection of different expression profiles, which nevertheless belong to the same cell type.

Collectively, the single-cell whole-transcriptome profiles contain information that can increase our understanding of the regulatory gene network in these cells (see Munsky et al. 10 for review). In addition, they provide an opportunity for a data-driven identification and definition of cell types, especially given the increasing throughputs in processing and analysis. This will add a new dimension to tissue sample analysis, allowing their description as a population of cells characterized by their cell types, similar to blood counts. We term these studies *population transcriptomics*. This approach has direct applications in neuroscience, where there is currently no comprehensive definition of cell types at the molecular level 1, or in cancer research, where tumors are heterogeneous and involve cancer cells as well as somatic cells that have a stable genome but are induced into pathological functions by the tumor cells. In stem cell research, it will allow a better understanding of the population dynamics within cell colonies or during trans-differentiation, where the role or effect of cell heterogeneity is still unclear.

Microfluidics is the study and the utilization of liquid flows in small volumes, and this miniature environment allows the reduction of reagent volume, avoids loss of sample or sample contamination, and provides a high throughput system for the integration of multiple functions in a *MicroTotal Analysis System* (µTAS) 11. Most microfluidics devices can be described as tiny assembly lines where reactions are carried out as the samples circulate from one specialized compartment to another through thin channels. These devices are embedded in carved platforms called *chips*, which are usually engineered by computer-aided design and produced using a system of photolithography, masks, and moulds 12 inspired by the methods of production of Micro Electro Mechanical Systems (MEMS). Chip designs can be classified according to the method of creating isolated compartments, with either solid borders materialized by microchambers, microwells, microvalves, etc., or fluid borders, in particular, microemulsions 13. While each cell comprises a compartment in itself, the methods for determining mRNA expression levels require cell lysis and, therefore, rely on the microfluidics device to isolate one cell per compartment.

**Figure 2:**
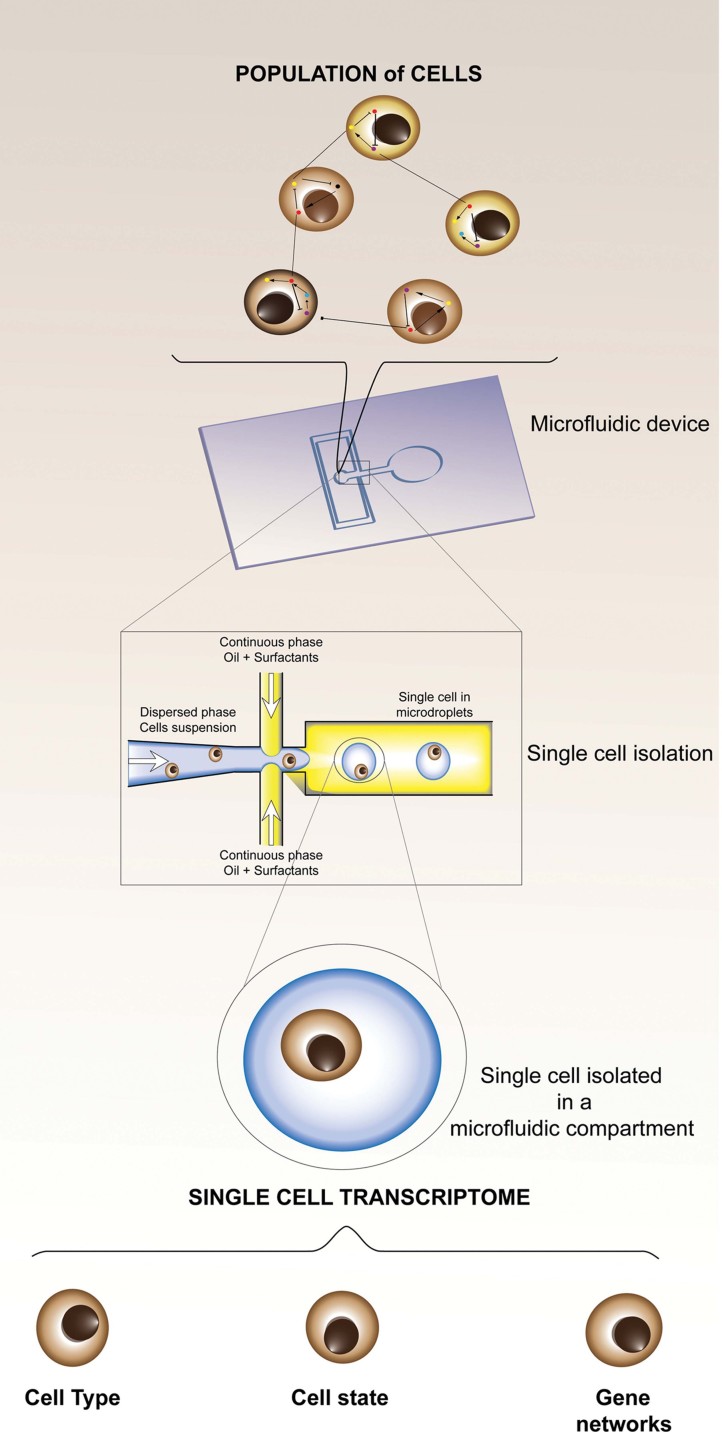
Droplet encapsulation of single cells with a microfluidic device. Single cells viewed as a complex heterogeneous population of individual cells interacting with each other and expressing different sets of transcripts. Single cells are loaded for encapsulation in a microfluidics device. The phase containing the cell suspension is squeezed by two oil flows that detach microdroplets containing single cells. Each droplet is a microreactor where reactions are confined. Single-cell transcriptome studies yield profiles to infer the cell type, the cell state, or the regulatory gene network active in the cell.

In this review we will describe how single cell transcriptome analysis using microfluidic formats opens the doors to understanding cell population structures. First, we will focus on microemulsions (Fig. 2), which promise a high throughput single cell analysis. Next, we will review the “all integrated” devices for single cell analysis of transcript expression levels. Finally, we will introduce new ways to investigate single cell transcriptomes and discuss the impact of high-throughput microfluidics.

## Microdroplets are tiny liquid compartments

Droplet microfluidics 14 refers to the formation and transport of liquid nano- to microdroplets in a non-miscible carrier medium such as oil. Water-in-oil emulsions provide a popular and scalable system to produce these picoreactors of very small volume. Because they are generated at high frequency (several kHz) 15, they offer opportunities for high throughput and single cell analysis. The oil (mineral, silicone, or fluorocarbon) that separates each droplet is non-miscible and inert. Surfactants prevent droplets from coalescing. Recently, fluorocarbon oils have gained popularity, thanks to the development of non-ionic, biocompatible surfactants 16, 17. Microdroplets can be formed by focusing oil and water flows (Fig. 2 and 18). Single cell encapsulation is not traumatic for cells and allows cell survey, cell screening, multicellular organism growth 19, or transfection 20.

Cells in suspension in the aqueous phase are encapsulated stochastically following a Poisson law 21. As a consequence, a large number of empty droplets are produced under conditions that prevent the encapsulation of two cells in the same droplet. To counter this drawback, different strategies have been adopted: inlet microchannel geometry design for self-organization of cells 22, on-demand laser driven drops 23, or improvement in droplet sorting. In the latter, after single cell encapsulation, microdroplets are sorted by shear-induced flow 21, fluorescence-activated dielectrophoresis (DEP) 24, or piezoelectric effect 25.

Compartmentalization can also be achieved by simple spatial separation. For instance, Lin et al. 26 created arrays of droplets covered by oil and separated with hydrophobic areas on a cover slip, requiring no special instruments. More sophisticated strategies also exist. Using a commercially available microdispersing instrument similar to inkjet printers, Liberski et al. 27 printed an array of droplets of medium, in which they injected cells by printing a smaller volume in the larger droplet medium. The droplets were covered by oil to prevent evaporation, but others achieved setups with no oil by maintaining a locally high hygrometry 28. Lastly, other water-in-oil confinements have been investigated, for instance with the *chemistrode* 29, where a succession of oil phases and aqueous “plugs” provides a temporal resolution, or the *SlipChip* 30, where channels materialized by the superposition of two carved plates can be reversibly converted into nanoliter wells.

## How to manipulate microdroplets?

Once the droplets are formed, it is difficult to introduce new reagents and carry out complex protocols. It is therefore necessary in some cases to break up the emulsions. To maintain the monoclonality of the reaction products after removing the oil phase, that is, to keep the single-cell resolution after opening the compartments, researchers have investigated two possibilities. In the first, the reaction products are bound to beads. The most prominent example is the 454 sequencing platform 31. Alternatively, reaction products or cells have been encapsulated in microgels, such as agarose 32 or polyethyleneglycol, gelated by polymerization with hyperbranched polyglycerol 33. After the reaction, the gel drops solidify, thus trapping the amplicons. To avoid diffusion of PCR products out of the clonal agarose beads, Leng et al. 34 conjugated the forward PCR primers directly with the agarose.

Given the stochasticity in gene expression levels, it is important that no artificial noise be added to the biological variations, which may be informative. Therefore, the single cell devices must avoid inducing stress pathways that are likely to cause the transcription of some genes or the degradation of some transcripts. In addition to radical solutions like pre-fixation, the microfluidic designs can perform cell lysis quickly after tissue dissociation while maintaining high throughput compared to hand picking. Table 1 summarizes some methods to snapshot the cellular state while preventing cells from expressing stress genes.

**Table 1:** Non-traumatic cell lysis methods

Methods	Techniques	References	Characteristics	Drawbacks
Optical	Laser-induced plasma formation	87, 88	Shock waves induce cell lysis	Need equipment
			Nanosecond to millisecond scale	
Chemical	Lysis buffer	89	Chemical disruption at 75°C of cell membrane	Enzyme denaturation in case of one step RT-PCR
			Powerful	
Electrochemical	Electric field	90	Electric field and emulsification agent	Special buffer conditions required

Multi-step reactions can be facilitated by delivery of new reagents for the next reaction steps. In microdroplet formats, reagents can be added by picoinjection or by merging droplets (Table 2), as demonstrated in the high-throughput single cell screening reported by Brouzes et al. 35, or in the microfluidic systems for digital DNA amplification and counting using rolling circle as shown by Mazutis et al. 36. Droplet fusion can be utilized not only for delivering contents; it can also be used for simple merging of droplets, for instance by electrocoalescence 37. Recently developed mesh-integrated arrays for merging and storage 38 offer the possibility to isolate single cell drops in picoliter compartments and to mix reagents for single cell-based assays. Incubation devices for cells or reactions are detailed in Table 3.

**Table 2:** Methods to mix reagents in microdroplets

Methods			Principe	Characteristics	References
Microdroplets merging	Passive	Decompression merging	Microchannel geometry change	Continuous phase drainage, first drops delay	18, 91–94
Active	Electrocoalescence	With electric field, modification of ionic charge of droplet interface	Droplets with opposite charge attracted	21, 95, 96
	Laser fusion	Localized heating close to the touching interfaces evacuates the surfactant molecules and oil film.	Fast and need equipment	18
Magnetic beads based fusion	With magnetic beads embedded in drops, move drops in order to merge them	Easy control of drops merging and allow reagent mixing, In oil in air microdroplets	89, 97
Picoinjection			Reagent is injected in a microdroplet with picoinjector. Pico injection is triggered with an electric field.	Precise, need electrodes for picoinjection control.	98

**Table 3:** Incubation systems

References	Characteristics
99	Microwave dielectric heating with indium alloy wire inserted into the PDMS chip
17	Long serpentine channel (144 µL)
100	“Picotiter array” using parallel channels, to monitor the growth rate of single cells
101	Immobilization in local storage areas
102	Immobilization in array, with capture rates above 90%. Reversion of flow allows droplets release
103	Large pillars-supported storage reservoir localized in the end of the device
24	External incubation, collecting droplets with a Pasteur pipette, and reloading them with a syringe
52	Delay lines made of elongated channels under reduced flow

Altogether, these microdroplets are a toolbox in which the usual molecular biology reactions performed with microtubes and micropipettes are being reimplemented at a much smaller scale for high-throughput single-cell analysis pipelines. But sometimes this approach, which causes some cells to be wasted, is not optimal. Proof-of-principle encapsulation devices often assume an unlimited input of cells, which is not realistic except in the cases of abundant primary cells or cultured cells. These experimental systems take the best from high throughput designs, but more focused designs are necessary when the cells of interest are determined in advance or are very limited in quantity, as in the case of early embryos, and therefore should not be lost. Devices using capture chambers instead of droplets still offer nanoliter-scale reactions, while reaching capture efficiencies as high as 5% 39. More complex combinations of technologies, with droplet sorting in the chips and cell pre-labeling (for instance by the injection or endocytosis of markers) might not be available in the short-term.

## Integrated microfluidics devices are like miniaturized assembly lines

**Figure 3:**
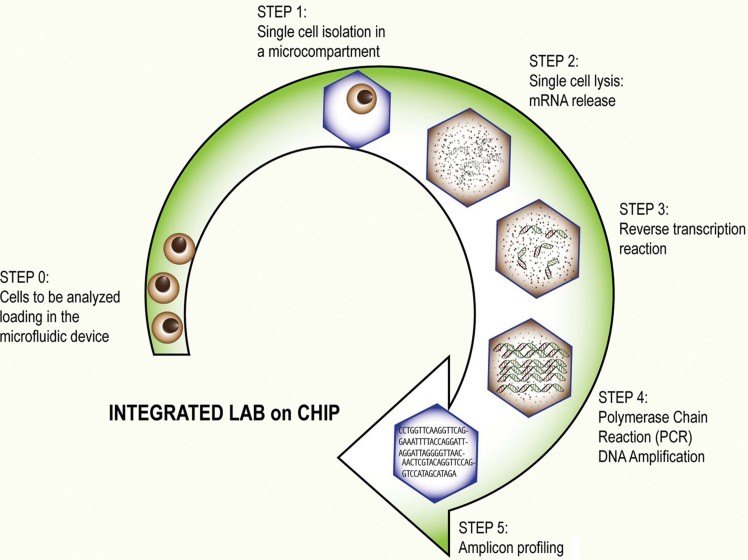
Steps to integrate in a device for single cell transcriptome profiling. In an all-integrated device, single cells are isolated in microcompartments (Step 1). Total mRNA is released by cell lysis (Step 2). In the next step, mRNAs are converted into cDNA by reverse transcription (Step 3). PCR amplification follows with on-board mechanisms for temperature cycling or external heating and cooling (Step 4). Finally the generated amplicons are collected and purified for sequencing (Step 5).

The construction of integrated devices for transcriptome analysis is challenging, as it requires the combination of the critical steps previously described (Fig. 3). Zhong et al. 40 used microchambers in which the mixing of reagents is easy, thus making the transition from RT to PCR very straightforward. They obtained a RNA to cDNA conversion yield of 54% in the device, compared to 12% with the same protocol executed in conventional tubes. Bontoux et al. 41 designed a two circular chamber device for *HPRT* and *GAPDH* RT-PCR amplification from a single cell. But attempts at whole-transcriptome RNA amplification by template-switching failed, most likely due to the lack of a purification step between RT and PCR; in the absence of purification, the template-switching oligonucleotides compete with the universal PCR primers, thus inhibiting the amplification reaction. Toriello et al. 42 assembled the most complete device, which includes cell selection in a nanoreactor, capture, lysis, reverse-transcription, PCR, purification of products, and separation.

By adjusting or producing components to fit their needs, multiple teams have assembled droplet-based devices of increasing complexity. It is expected that transcriptome analysis is also likely to be achieved using this compartmentalization strategy. Multiple groups have already demonstrated RNA capture followed by cDNA synthesis 40, 43, 44 and PCR amplification on the microdroplets format 45–47.

Transcriptome analysis includes the study of non-coding RNAs such as micro-RNAs, and White et al. 39 estimated miRNA and mRNA molecule counts by qPCR in K562 cells using a multichamber design where the cells were captured, washed, and lysed, and cDNAs synthesized and then amplified by PCR with gene-specific primers. In addition to easy reagent flow control, microchambers also allow parallelization of reactions, and can process up to 300 cells per chip.

While already a multi-step protocol, PCR itself is a building block for more complex workflows. A high-throughput single copy genetic amplification (SCGA) process has been developed by Kumaresan et al. 48. In this process, cells or target DNA are encapsulated in nanoliter droplets containing functionalized beads 49. The PCR products are bound to the beads, and analyzed in flow cytometers. With a throughput of one million droplets per hour as a design goal, Zeng et al. 50 reported the conception of Microfabricated Emulsion Generator Arrays (MEGA), in which single cells are encapsulated in droplets containing beads coated with forward PCR primers, and fluorescently labeled reverse PCR primers. After cell lysis and PCR, the beads are bound with fluorescently labeled amplicon if the PCR was positive. The result of the experiment is then read by counting in a flow cytometer, and the beads exhibiting fluorescent labels are chosen for each target gene.

Multiplexing of devices is achieved in a straightforward manner by replicating a building block on the lab chip. This strategy fits particularly well in cases where the reagents or the starting materials are pre-arranged in standard formats such as the 96-well plate. But alternatives are needed when aiming at higher orders of magnitude. By preparing a reagent droplet library in which each droplet contains a specific primer pair and these are fused to droplets containing single-cells, Tewhey et al. 51 developed an enrichment device for large-scale targeted sequencing. Incubation is conducted outside the device in a thermocycler and the products are recovered by breaking the emulsion with a destabilizer solution. Another modular strategy, with two devices connected by an incubation channel functioning as a delay line 52, was described by Agresti et al. 53. This featured cell encapsulation, drop incubation, and sorting for directed evolution. In another screen, Brouzes et al. 35 fused a library of single-chemical droplets to droplets containing single human monocytic U937 cells. The chemical contents of the merged droplets were tracked by optical coding with a combination of fluorochromes, a strategy that might be employed later in a different context, for instance to record treatments, spatial origin or temporal series.

## Single-cell expression levels measured as transcript counts

Devices that can prepare large numbers of single cells for gene expression analyses open the way to a new representation of the cell's transcriptome. With current studies of cell mixtures, transcript expression levels are measured relative to each other using methods such as quantitative PCR, microarrays, cDNA sequencing (RNA-Seq, etc.) or in situ hybridization. Consequently, our tools and concepts are centered on these approaches in which a significant fold-change will be a positive result. However, relative expression levels are an incomplete readout of a simpler parameter: the number of transcripts present in a sample.

Single-cell whole-transcriptome counting technologies aim at absolute precision. One key challenge is to prevent loss of templates. Losses seriously complicate the interpretation of the data since the absence of signal would not exclude that the cells were expressing the RNA. Two strategies to avoid these losses differ in their trade-off between complexity and sensitivity. Single-molecule sequencing 54 provides direct counts but offers no protection against losses. The aim of this approach is, therefore, to simplify the protocol as much as possible, for instance by sequencing RNA by synthesis instead of using a cDNA intermediate 55. On the other hand, in methods in which the transcripts are amplified, usually as cDNAs, passive adsorption and similar losses will not cause a transcript to become undetectable. However, expression levels may be biased by the amplification unless the cDNAs that originate from the same mRNA molecule can be recognized and considered as a single count. This has been done by Kivioja et al. 56 with the introduction of unique molecule identifiers (UMIs). Alternatively, the use of random primers in the reverse transcription will produce a collection of cDNAs where the random priming site itself can be considered as a unique molecular identifier, such as in CAGEscan 57, provided that there is no strand displacement. This strategy could be generalized to any semi-anchored transcriptome analysis technology, for instance when the anchor is the RNA 3′-end, via oligo-dT priming or linker ligation.

## qPCR and imaging contribute independent observations

Before reaching a critical mass and becoming well established, sequence-based data will need to be confirmed by other methods. In particular, it is still unclear how many RNA molecules are expected in single cells. Beyond the effects of stochastic expression, surprisingly large variations can be found in subpopulations of cells that would otherwise be considered of the same type. Molecule counting has been approximated by quantitative PCR using a titration curve. For instance, Ståhlberg et al. 58 analyzed single astrocytes and single neurosphere cells by qPCR (50 cycles with optimized primers) and found two sub-populations distinguished by their low or high expression of marker genes, where the highest counts exceeded 40,000 for the *Vimentin* or *GFAP* transcripts. Taken together, these two subpopulations could explain more complex expression patterns as the sum of two simpler lognormal-style distributions. Molecule counting has also been implemented by another application of microfluidics, digital PCR 59, 60. In this droplet-based embodiment, up to one million reactions are parallelized, leading to an average error of 15%, to be compared with qPCR, which produces a quantitative error of 100% in just a single cycle (as it doubles the product once per cycle) 61.

Sequencing methods and qPCR have common limitations. In particular, they are not in situ and are, therefore, unable to distinguish nascent RNAs from mature RNAs unless pulse-labeling or run-on methods are implemented 62. This would be challenging in microfluidics devices. Second, they are inherently limited by the incompleteness of the reverse-transcription step 63. Therefore, transcript counting by imaging is an important complementary technique, providing a technologically independent line of evidence to the sequencing and qPCR-based measurements. This technology is being actively developed and is progressing in throughput 4, 64.

Imaging-based analysis of mRNA expression levels is amenable to microfluidics miniaturization. By confining single cells in channels and tracking them across cell divisions, Rowat et al. 65 studied the diversity of modes of expression in yeast. Using three genes as models and GFP fusions as a reporter system, they could demonstrate constitutive (Rps8b), inherited (Pho84), and heterogeneous (Hsp12) expression modes. This diversity has been reported earlier and is often modeled as *bursts* of transcription. However, many of the pioneering studies were focused on a small number of genes. Using a device for high throughput imaging by batches of 96, Taniguchi et al. 66 covered the whole genome and reported no correlation between protein and RNA levels in *Escherichia coli*. Importantly, Taniguchi et al. derived a model where Gamma distribution parameters were interpreted as transcription rate and protein burst size. This lack of correlation was also observed by Raj et al. 7, who noted that while the *RNA polymerase II* gene was expressed in bursts, the bursts in other genes were not synchronous, suggesting that the transcription noise is buffered at the protein level. Indeed, using a destabilized GFP, Raj et al. observed correlation between protein and mRNA levels, which could not be observed with the usual, more stable GFP. Other mechanisms also decouple the expression levels of proteins and mRNAs. For instance, mRNA can be stored in P-bodies and stress granules directly after transcription 67.

Methods for highly multiplexed transcript counting by imaging have also been developed. For instance, NanoStrings 68 were used with single cells by Khan et al. 69 to demonstrate that the olfactory enhancers function to increase the number of cells expressing transcripts, as opposed to the levels of transcript expression in each cell. This work also showed that hybridization-based technologies can reach a high stringency, since the authors could distinguish 577 different olfactory receptors despite strong homologous sequences.

## Cells that look similar can contain different amounts of transcripts

While studying the distribution of transcript expression levels, it will be essential to ensure that technical noise is not added to the biological variations. In particular, a high confidence level is needed to distinguish between the true absence of a transcript and the failure of its detection. Indeed, Bengtsson et al. 70 showed that RT-PCR noise is stronger than biological variations for transcripts present in less than 100 mRNA or 20 cDNA copies. They also reported striking differences in reverse-transcription efficiencies, ranging between 2% and 99%. Miniaturization has the potential to mitigate this problem. For instance, in the device of Zhong et al. 40 the minimal detectable number of molecules was four, compared to 17 in a bulk assay. More recently, Reiter et al. 5 showed that in lymphocytes, the noise associated with reverse-transcription was far greater than the technical noise caused by the presence of cell debris in the mixture, which suggests that reaction volumes could be reduced in micro-compartments. Altogether, the methods based on reverse-transcription will need to be carefully calibrated by comparing them with the measurements based on imaging.

Zenklusen et al. 9 used 5′ probes to study transcription initiation in *S. cerevisiae* cells. Nascent transcripts are detected as a nuclear signal, of which the intensity is a multiple of the single spots in the cytoplasm. They estimated ∼60,000 mRNAs per cell, which is 3–6 times higher than previous estimates. In this study of three house-keeping genes, less than 10% of the cells did not contain the target RNA, and the data had a Poisson distribution. Study of the *MDN1* gene indicated pauses longer than one minute between transcription initiations. The data fits models of bursts but it is important to note that other models were also compatible. The study of other genes suggests that not all genes are transcribed in bursts. For instance, *POL1*, regulated by cell cycle, did not show bursts as most cells did not contain more than one nascent RNA, in contrast to the SAGA + TATA-controlled gene *PDR5*.

Unlike relative expression levels, expression counts are dramatically modified at each cell division, where they would be halved if the division were symmetric. However, with the exception of very tightly controlled molecules such as chromatids, most molecules are not equally segregated between daughter cells. Huh and Paulsson 71 have demonstrated that models of random partition noise can also predict stochastic expression counts, that are often solely modeled as the outcome of transcription bursts in the literature. Moreover, they also show that molecular mechanisms such as aggregation or sub-cellular localization in vesicles can further increase that noise. In fast-dividing cells, partition noise of low-expressed upstream regulators might also explain the stochasticity of expression. In addition to dilution by cell division, levels of RNAs are also decreased by degradation. This can also be studied by imaging, for instance by comparing the output of 5′ and 3′ probes 7.

Heterogeneity within a population can also be caused by other mechanisms, such as ageing. Taken on more than a generation, all cell divisions are potentially asymmetric, as one daughter cell may inherit a half where the molecules or the organelles have been synthesized at an earlier generation and therefore had more time to accumulate damage. In line with this hypothesis, Wang et al. 72 showed in *E. coli* that cells do not die by exponential decay, but rather, the cells that inherit the halves that have been passed down over many generations are distinguishable from others by their higher probability to die. Altogether, cell division has direct implications on the heterogeneity of the transcriptome.

## Perspectives

The microdroplets format offers high throughput opportunities and will probably become one of the most powerful tools for profiling a population of cells with single-cell resolution. In an all-integrated device, one-step lysis and cDNA synthesis will require thermostabilization 73. Integrated instruments will also face the difficulties of merging droplets, and controlling reaction temperature, while avoiding leakage of small molecules from droplets 74. Commercial solutions have a strong incentive to overcome the Poisson encapsulation and the resulting 70% of empty drops. Finally, the collection of microdroplets for amplicon sequencing will require emulsion purification 32. Chamber-based technologies, which are maturing faster and now arriving on the instrument market, will remain an excellent complementary technology for samples delivering a small number of cells, the transcriptomes of which will be compared to cell population models solidly established by the comprehensive reference data that will be produced in the coming years.

These models will also have to integrate multiple sources of heterogeneity within a cell population, from the most obvious such as the cell cycle, to less tractable factors such as a cell's position within a tissue. There is currently no high-throughput technology for whole-transcriptome analysis that preserves spatial coordinates, and while pioneering studies can be conceived in transgenic models expressing spatially controlled reporter genes, strategies must be prepared for the study of human tissues, where this method will not be available. This calls for the joint application of microfluidics and other methods achieving single-cell resolution, but that preserve the tissue integrity, or for labeling strategies that allow us to record and read the spatial information. The relevance of spatial information has been highlighted by Snijder et al. 75, who studied HeLa cells infected by five different viruses, and showed that the cell's position in a culture determines the activity of some pathways, such as focal adhesion kinase, which in turn determines the susceptibility to viruses. Combinations of imaging and amplification techniques, such as RT-LAMP 76 or RT-SmartAmp 77, have not yet been explored for studying expression in situ.

The study of the diversity of cell states can be strengthened by the collection of other data produced at single-cell resolution. For instance, Kim et al. 78 measured the distribution of ATP concentration in cells immobilized on a trapping array. Boitard et al. 79 monitored the metabolic activity of single yeast cells encapsulated in droplets, using osmotic exchanges with neighboring empty droplets as a readout for fermentation of glucose. Kelbauskas et al. 80 measured oxygen consumption rates using extracellular optical sensors in microwells of subnanoliter volume. Incidentally, an indirect measure of the number of ribosomes per cell may be provided by transcript sequencing methods, which usually focus on mRNAs and discard rRNAs to reduce the cost of sequencing, but which may now have to skip that step to achieve miniaturization.

### What are the current limits?

The cost per read count limits the throughput of analysis. However, there are incentives for the analysis of large numbers of cells. First, high-throughput designs are simple since they do not require careful handling of a limited amount of cells, that is, they tolerate losses. Second, identifying cell types may not be enough to represent a cell population. For instance, within a cell type, it may be important to determine if the cells are cycling or resting. The definition, and therefore the number, of known cell types may change in the next few years as a result of single-cell transcriptomic studies. It is therefore difficult to foresee an optimal number of sequence reads. However, once population definitions are well seeded by comprehensive databases of single-cell transcript counts, there may be a possibility that only a shallow sampling of each cell's transcriptome will be enough to classify them and establish their population structure. This suggests that we may need to aim for high throughput sequencing in the order of a million cells per cell type.

Such a high throughput calls for the development of new computer representations of the results, which would allow us to share, archive, retrieve, and re-analyze experimental results at a sustainable cost. This problem is strikingly similar to the challenge of working with billions of personal genome sequences, and may be solved by representing each cell by its difference with a close reference. This again highlights the importance of seed projects defining cell types in terms of model distributions of transcript counts. Following transcript variants, such as splice isoforms, will also be challenging both from the molecular biology and the computational points of view.

Beyond stochastic variations, individual differences between cells of the same type can have significant functional consequences. For example, whether a cell was captured at the moment when it was sending a signal or not. This suggests that the whole transcriptome, and not just the transcripts that are sufficient to determine a cell's identity, must be considered in order to understand a cell's activity. However, some methods that are apparently whole-transcriptome are still restricted in their scope, in particular when the mRNAs are captured or reverse-transcribed with oligodeoxythymidine primers, as up to a third of the mRNA may be non-polyadenylated 81, 82. Other non-polyadenylated RNAs have attracted considerable attention: the micro RNAs. As long as RNA samples could be split in long and short fractions, there was no incentive to develop a universal protocol to sequence them together with mRNAs, but with single-cells as the target, there is no other choice. Template-switching was used 83 to prepare miRNA libraries from bacteria and also to study mRNAs 6, 57, 84, and therefore is a good candidate for such an universal protocol.

## Conclusion

Microfluidic technology is on track to be a serious provider of single-cell transcriptomes. With miniaturization, the decrease of reaction volumes brings several advantages. First, the concentration of the samples, and therefore the yield, can be dramatically increased 40. Second, miniaturization allows for very high throughputs, in the order of a million cells per hour 50. Third, some physical phenomena, including diffusion or heat transfer, are quickly completed in small volumes, accelerating the transition between protocol steps. The unit of single-cell whole-transcriptome measurements will be RNA counts 85, providing a snapshot of a cell's contents, which integrates production by the genome, degradation and dilution through cell division.

Cells can be further divided into smaller compartments (e.g. nuclear and cytoplasmic) or into compartments produced by them (e.g. vesicles and exosomes). Secreted exosomes, for instance, are already the subject of some microfluidic-assisted transcript expression analysis 86. Nevertheless, because cells are a natural functional unit, single-cell whole-transcriptome analyses are likely to become the bulk of the small-sample studies, aided by microfluidics technologies that will simplify their handling, thus increasing reproducibility and providing a high throughput, while aiming at costs similar to or lower than classical analyses of mixtures. Single-cell resolution will add a new dimension to biological sample analysis, *population transcriptomics*.
